# The Role of Serratomolide-like Amino Lipids Produced by Bacteria of Genus *Serratia* in Nematicidal Activity

**DOI:** 10.3390/pathogens11020198

**Published:** 2022-02-01

**Authors:** Catarina Marques-Pereira, Diogo Neves Proença, Paula V. Morais

**Affiliations:** Department of Life Sciences, Centre for Mechanical Engineering, Materials and Processes, University of Coimbra, Calçada Martim de Freitas, 3000-456 Coimbra, Portugal; catarina.103@gmail.com (C.M.-P.); pvmorais@uc.pt (P.V.M.)

**Keywords:** *Serratia*, serratomolides, serrawettin W1, serrawettin W2, pine wilt disease, pinewood nematode, *Bursaphelenchus xylophilus*

## Abstract

*Bursaphelenchus xylophilus*, also known as pinewood nematode (PWN), is the pathogenic agent of pine wilt disease (PWD), which affects pine trees around the world. Infection spread globally through international wood commerce and locally by vector beetles, threatening the wood world economy. As climate changes, more countries are becoming susceptible to PWD and, to prevent disease spread and limit economic and ecological losses, better knowledge about this pathogenic agent is needed. *Serratia* strains, present in the endophytic community of pine trees and carried by PWN, may play an important role in PWD. This work aimed to better understand the interaction between *Serratia* strains and *B. xylophilus* and to assess the nematicidal potential of serratomolide-like molecules produced by *Serratia* strains. Serrawettin gene presence was evaluated in selected *Serratia* strains. Mortality tests were performed with bacteria supernatants, and extracted amino lipids, against *Caenorhabditis elegans* (model organism) and *B. xylophilus* to determine their nematicidal potential. Attraction tests were performed with *C. elegans*. Concentrated supernatants of *Serratia* strains with serratamolide-like lipopeptides were able to kill more than 77% of *B. xylophilus* after 72 h. Eight specific amino lipids showed a high nematicidal activity against *B. xylophilus*. We conclude that, for some *Serratia* strains, their supernatants and specific amino lipids showed nematicidal activity against *B. xylophilus*.

## 1. Introduction

Pine forests face a global threat, pine wilt disease (PWD), caused by pinewood nematode (PWN) *Bursaphelenchus xylophilus* which is carried from tree to tree by *Monochamus galloprovincialis* vector beetles [1]. *B. xylophilus* is native to North America and was first reported in Japan, where it rapidly caused a major pine forestry catastrophe [2]. Subsequently, it was spread to other Asian countries as China, Korea and Taiwan, causing severe economic damages [3]. PWN was later reported in Nigeria and Mexico [4,5]. Portugal was the first European country to detect PWD; PWN was detected in 1999 [6] and its vector *M. galloprovincialis* was confirmed in 2001 [7]. The *B. xylophilus* is listed as a European and Mediterranean Plant Protection Organization (EPPO) A2 quarantine pest in the European Union. In Europe, until this date, PWN was only reported in Portugal, currently affecting all of the mainland [8] and Madeira Island [9]; a few detections in Spain were soon after considered eradicated [10]. De la Fuente and Saura (2021) modelled the spatiotemporal patterns of future PWN natural spread in the Iberian Peninsula and found that, in the absence of effective containment measures, the PWN will spread naturally to the entire Iberian Peninsula, including the Pyrenees, where it would find a gateway for spread into France [11].

Phylum Nematoda includes five major taxonomic groups, involving *Caenorhabditis elegans* and *B. xylophilus* [12]. *C. elegans* was the first multicellular organism with a complete genome sequence available and continues to be considered an excellent model animal [13,14]. Plant pathogens such as PWN possess the ability to produce enzymes, such as cellulases, to degrade cell walls and penetrate and migrate over plant tissues [15]. The life cycle of PWN and PWD development have been addressed by several reviews [3,16,17]. Briefly, PWN is carried by insect vector *Monochamus* spp. inside their tracheal system and on their body surfaces. The insect vector is attracted by healthy young pine shoots and releases the nematodes during maturation feeding. PWN carried by vectors enter through feeding wounds and multiply in resin canals, attacking epithelial plant cells. The insect vector is later attracted by dead or dying trees for oviposition; during larvae development, *B. xylophilus* feeds on blue stain fungi. Before the adult insect vector emerges from the pupal chamber, the nematodes are attracted, enter the callow adult tracheal system and restart the disease cycle. After only three weeks of infection, trees start to show disease visual symptoms and weaker defense mechanisms [18]. There is no current way to control PWN after infection; damaged trees must be removed and destroyed. Insecticides against vectors are still in used in South Korea [19], but no longer in Japan, and were not used in Portugal. By infecting pine trees, PWN interacts with their endophytic microbial community [20], carrying its own bacteria [16]. In Portugal, bacteria associated with PWN belong mostly to the genera *Pseudomonas, Burkholderia, Enterobacteria, Serratia, Ewingella, Pantoea* and *Erwinia* [21]. Bacteria associated with PWD may be pathogenic and carried by PWN or endophytic and activated by *B. xylophilus* presence through a mutualistic effect [22,23]. Endophytic bacteria, in order to provide protection against plant pathogens and to promote plant growth, produce secondary metabolites such as siderophores (iron chelators and plant growth promoters), and enzymes as lipases [16,21,24,25]. Previous studies concluded that bacteria associated with PWN and the comprising endophytic community showed nematicidal activity and may contribute to the defense of host trees [26]. Strains from genus *Serratia*, present in the endophytic community and carried by PWN, were already reported as pathogenic to the PWD vector, *Monochamus* [27], and revealed an active nematicidal effect towards PWN [26]. These findings suggest that *Serratia* strains may help in future PWD management.

In order to colonize different surfaces, *Serratia* strains produce serratomolide biosurfactants, extracellular lipopeptides able to reduce surface tension and enhance flagellum spread growth [28]. Three different serratomolide lipopeptides were described with complex structures and synthesized by non-ribosomal enzymatic processes: serrawettin W1, a cyclodepsipeptide [29], serrawettin W2, a surface-active exolipid [30] and serrawetin W3 [31]. Biosynthesis of serrawettin W1 is thought to occur by condensation of two molecules of serratamic acid (D-3-hydroxydecanoyl)-L-serine [30], which is dependent on PPTase (4′-phosphopantetheinyl transferase). The serrawettin W1 gene (*swrW*) possesses 4476 bp, presents a large open reading frame (ORF) and has condensation, adenylation, thiolation and thioesterase domains in functional order [32]. Serrawettin W2 is a cyclic peptide containing a single fatty acid (3-hydroxydecanoic acid) and five amino acids (Leucine-Serine-Threonine-Phenylalanine-Isoleucine). This lipopeptide contributes specifically to surface bacterial translocation and has shown antimicrobial activity against many bacteria and fungi, as well as antitumor activity against Hela cells [30]. The serrawettin W2 biosynthetic gene (*swrA*) showed an architecture composed of five modules, each with a condensation, adenylation, and thiolation domain. Module 5 possesses an additional thioesterase (TE) domain. Biosynthesis is dependent on PPTase [33] and a hybrid polyketide synthase (PKS-NRPS) gene cluster was found to be involved in this process in *S. surfactantfaciens* YD25 [34]. The genome analysis of 84 publicly available genomes of the genus *Serratia* showed four genes common to all serrawettin gene clusters encoding a CTP (cytidine triphosphate) synthase, glyoxalase/bleomycin resistance protein/dioxygenase, LrgA family protein, and LrgB family protein, highlighting their essential potential in the serrawettins biosynthetic process [35].

Here, we aim to understand better the role-played in PWD by some *Serratia* strains and by its serratomolide-like lipopeptides in order to assess their nematicidal potential towards PWN. The presence of serrawettin genes was determined in nineteen selected *Serratia* strains from the University of Coimbra Bacteria Culture collection (UCCCB). Bacteria-nematode interactions were assessed through attraction and mortality tests with bacterial strains, respective supernatants, and amino lipids extracted from *Serratia* strains able to produce serratomolides. Attraction tests analyzed the olfactory ability of nematodes to discriminate between odors and to modify their behavior through olfactory learning. Nematicidal activity of supernatants, concentrated supernatants, and extracted amino lipids were performed.

## 2. Results

### 2.1. Amplification, Purification and Sequencing of swrW and swrA Genes

Nineteen bacterial strains were screened by PCR for the presence of *swrW* and *swrA* genes since these genes are the key genes to all serrawettin biosynthetic gene clusters; however, each *Serratia* strain showed only one type when present [35]. The PCR products corresponding to the expected gene-fragments sizes were sequenced and compared with known serrawettin genes. BLASTn analysis revealed that *Serratia* strains Arv-22-2.5c, Arv-22-2.6, Arv-29-3.9, NBRC 102599^T^ and AS13 carried the *swrW* gene and that *Serratia* strains A88copa7 and A88copa13 carried the *swrA* gene (Table 1). Both genes were not detected on the remaining *Serratia* strains after PCR amplification and gene sequencing.

### 2.2. Lipopeptides Extraction and Purification

**Figure 1:**
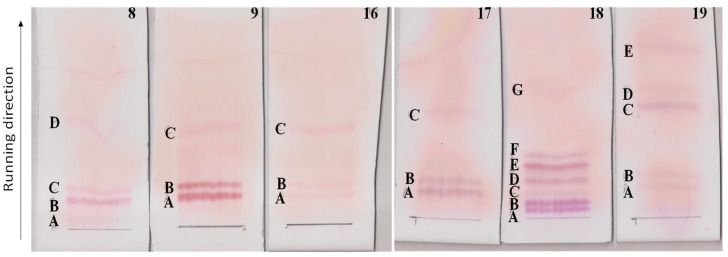
TLCs of polar lipids from supernatants of *Serratia* strains Arv-22-2.5c (TLC 8), Arv-22-2.6 (TLC 9), A88copa7 (TLC 16), A88copa13 (TLC 17), NBRC 102599^T^ (TLC 18) and AS13 (TLC 19). Positive amino lipids were spotted with ninhydrin solution, colored in pink.

After a 24 h growth in CAA medium to promote secundary metabolits production [26], *Serratia* strains Arv-22-2.5c, Arv-22-2.6, A88copa7, A88copa13, NBRC 102599^T^ and AS13 with serratomolides genes, the OD_600_ were observed (Table S3) followed by lipopeptides extraction and purification. Strain Arv-29-3.11b was not further analyzed since it was not able to recover after plate streaking.Thin layer chromatography (TLC) of polar lipids from each *Serratia* strain growth revealed four amino lipid spots on *Serratia* strain Arv-22-2.5c (8A, 8B, 8C, 8D); three on *Serratia* stains Arv-22-2.6 (9A, 9B, 9C), A88copa7 (16A, 16B, 16C) and A88copa13 (17A, 17B, 17C); seven on *Serratia* strain NBRC 102599^T^ (18A, 18B, 18C, 18D, 18E, 18F, 18G) and five on *Serratia* strain AS13 (19A, 19B, 19C, 19D, 19E) (Figure 1).

The TLC profiles of the different strains share band staining as amino lipids. Moreover, the amino lipids profiles were not directly related with the serrawitin genes detected (*swrW* or *swrA*).

### 2.3. Mortality Tests in C. elegans

**Figure 2:**
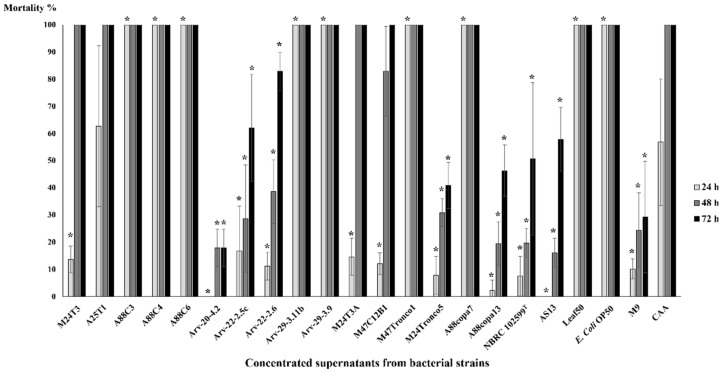
*C. elegans*’ mortality tests with concentrated supernatants of bacterial strains and M9 and CAA media as controls. Supernatants were concentrated by vacuum and evaporation at low temperatures (−40 °C) after a 24 h growth of *Serratia* strains M24T3, A25T1, A88C3, A88C4, A88C6, Arv-20-4.2, Arv-22-2.5c, Arv-22-2.6, Arv-29-3.11b, Arv-29-3.9, M24T3A, M47C12B1, M24Tronco5, A88copa7, A88copa13, NBRC 102599^T^, AS13 and Leaf50, and two controls *Pseudomonas* sp. M47Tronco1 and *E. coli* OP50. The number of dead nematodes was assessed at 24 h, 48 h and 72 h after incubation with concentrated supernatants. Each test was performed in triplicate. After one-way ANOVA, the comparison for mortality between the different strains was statistically significant for all the time points (24 h: F(21, 44) = 67.62, *p* < 0.001; 48 h: F(21, 44) = 76.04, *p* < 0.001; 72 h: F(21,44) = 25.80, *p* < 0.001). Results that showed statistical differences on the post hoc Tukey’s Test at *p* < 0.05 between strain and CAA medium are marked with (*). The lines above the bars represent standard deviation.

**Figure 3:**
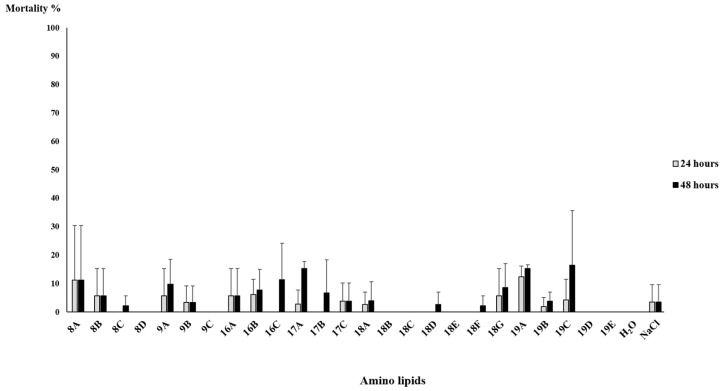
*C. elegans*’ mortality tests with amino lipids eluted in 0.1 M NaCl solution and H_2_O and 0.1 M NaCl solution as negative control. Amino lipids were extracted from supernatants of a 24 h growth of *Serratia* strains Arv-22-2.5c (amino lipids 8A, 8B, 8C and 8D), Arv-22-2.6 (amino lipids 9A, 9B and 9C), A88copa7 (amino lipids 16A, 16B and 16C), A88copa13 (amino lipids 17A, 17B and 17C), NBRC 102599^T^ (amino lipids 18A, 18B, 18C, 18D, 18E, 18F and 18G) and AS13 (amino lipids 19A, 19B, 19C, 19D and 19E). The number of dead nematodes was assessed at 24 h and 48 h after incubation with amino lipids. Each test was performed in triplicate. After one-way ANOVA, the comparison for mortality between the different amino lipids was not statistically significant for all the time points (24 h: F(26, 54) = 0.959, *p* = 0.534; 48 h: F(26, 54) = 1.326, *p* = 0.189). The lines above the bars represent standard deviation.

Mortality tests were performed to assess the nematicidal properties of bacterial supernatants. Ten *C. elegans* were used in each test in order to evaluate which strains have nematicidal properties against them. Mortality tests were performed in triplicate with concentrated supernatants (Figure 2) and extracted amino lipids (Figure 3).

Only concentrated supernatants from *Serratia* strains A88C3, A88C4, A88C6, Arv-29-3.11b, Arv-29-3.9, A88copa7, Leaf50, *Pseudomonas* strain M47Tronco1 and *E. coli* OP50 were able to kill 100% of *C. elegans* after 24 h, with statistical significance (Figure 2, Table S4). Concentrated supernatants from *Serratia* strains M24T3, A25T1, M24T3A and M47C12B1 showed 100% dead *C. elegans*. This was also visible for control CAA at the same time.

Mortality tests in *C. elegans* with extracted amino lipids revealed no statistical difference when compared to the 0.1 M NaCl solution (the solution used to resuspend TLC fractions); overall mortality rates were less than 15% (Figure 3). The *Serratia* strain A88copa13 supernatant that was previously identified as nematicidal for *B. xylophilus* [26] because it produced a serine protease to the supernatant was considered as a positive control for the experiment. Nevertheless, the strain supernatant was unable to have a nematicidal effect against *C. elegans*. *E. coli* OP50 with an OD_600_ = 0.4 was able to kill all nematodes in 24 h.

### 2.4. Mortality Tests in B. xylophilus

**Figure 4:**
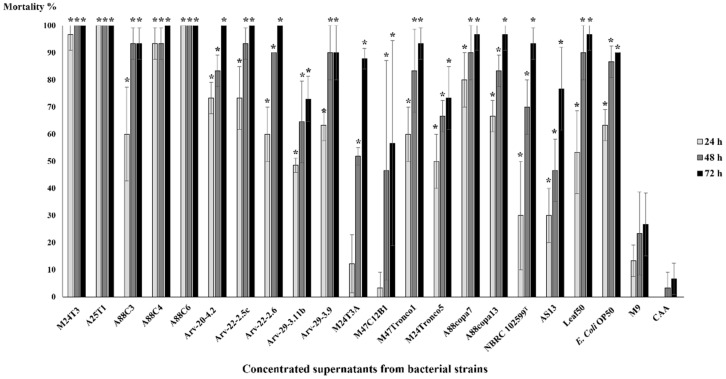
*Bursaphenchus xylophilus*’ mortality tests with concentrated supernatants of bacterial strains and M9 and CAA media as control. Supernatants were concentrated by vacuum and evaporation at low temperatures after a 24 h growth of *Serratia* strains M24T3, A25T1, A88C3, A88C4, A88C6, Arv-20-4.2, Arv-22-2.5c, Arv-22-2.6, Arv-29-3.11b, Arv-29-3.9, M24T3A, M47C12B1, M24Tronco5, A88copa7, A88copa13, NBRC 102599^T^, AS13 and Leaf50, and controls *Pseudomonas* sp. M47Tronco1 and *E. coli* OP50. The number of dead nematodes was assessed at 24 h, 48 h and 72 h after incubation with concentrated supernatants. Each test was performed in triplicate. After one-way ANOVA, the comparison for mortality between the different strains was statistically significant for all the time points (24 h: F(21, 44) = 31.29, *p* < 0.001; 48 h: F(21, 44) = 14.31, *p* < 0.001; 72 h: F(21,44) = 17.27, *p* < 0.001). Results that showed statistical differences on the post hoc Tukey’s test at *p* < 0.05 between strain and CAA medium are marked with (*). The lines above the bars represent standard deviation.

Mortality tests were performed to assess the nematicidal properties towards PWN of bacterial supernatants. Ten *B. xylophilus* in each test were used in order to evaluate which strains have nematicidal properties against this nematode (Figure 4, Table S5).

*B. xylophilus*’ mortality tests with concentrated supernatant revealed that this nematode is susceptible to *Serratia* strains. Almost every concentrated supernatant, when compared with negative control, showed statistical differences after 24 h, namely concentrated supernatants from *Serratia* strains M24T3, A25T1, A88C3, A88C4, A88C6, Arv-20-4.2, Arv-22-2.5c, Arv-22-2.6, Arv-29-3.11b, Arv-29-3.9, M24Tronco5, A88copa7, A88copa13, NBRC 102599^T^, AS13 and Leaf50, *Pseudomonas* strain M47Tronco1 and *E. coli* OP50. In these mortality tests, strains Arv-22-2.5c, Arv-22-2.6, Arv-29-3.9, A88copa7 and A88copa13, which have serrawettin genes, were able to kill more than 60% of *B. xylophilus*. After 48 h and 72 h, concentrated supernatants from all strains showed statistical differences when compared to control (CAA medium); all strains had a nematicidal activity against PWN. *Serratia* strains A25T1 and A88C6 were the only ones able to kill all *B. xylophilus* at 24 h (Figure 4).

**Figure 5:**
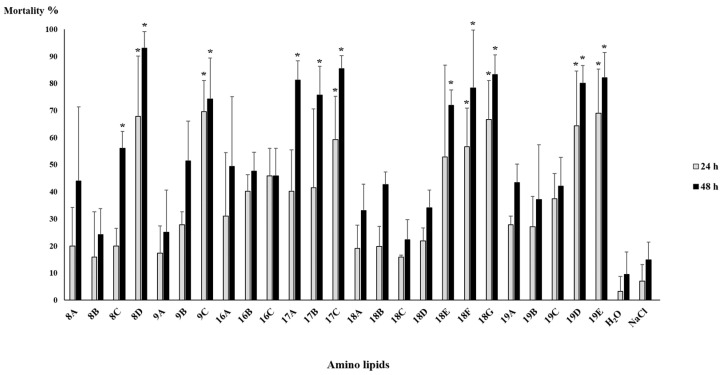
*B. xylophilus*’ mortality tests with amino lipids eluted in 0.1 M NaCl solution and H_2_O and 0.1 M NaCl solution as negative control. Amino lipids were extracted from supernatants of a 24 h growth of *Serratia* strains Arv-22-2.5c (amino lipids 8A, 8B, 8C and 8D), Arv-22-2.6 (amino lipids 9A, 9B and 9C), A88copa7 (amino lipids 16A, 16B and 16C), A88copa13 (amino lipids 17A, 17B and 17C), NBRC 102599^T^ (amino lipids 18A, 18B, 18C, 18D, 18E, 18F and 18G) and AS13 (amino lipids 19A, 19B, 19C, 19D and 19E). The number of dead nematodes was assessed at 24 h and 48 h after incubation with amino lipids. Each test was performed in triplicate. After one-way ANOVA, the comparison for mortality between the different amino lipids was statistically significant for all the time points (24 h: F(26, 54) = 5.709, *p* < 0.001; 48 h: F(26, 54) = 11.43, *p* < 0.001). Results that showed statistical differences on the post hoc Tukey’s test at *p* < 0.05 between amino lipid and NaCl solution are marked with (*). The lines above the bars represent standard deviation.

Mortality tests performed with extracted amino lipids in *B. xylophilus* (Figure 5) revealed higher mortality rates when compared to *C. elegans* (Figure 3). When compared with the 0.1 M NaCl solution, amino lipids 8D from *Serratia* strain Arv-22-2.5c, 9C from *Serratia* strain Arv-22-2.6, 17C from *Serratia* strain A88copa13, 18F and 18G from *Serratia* strain NBRC 102599^T^, 19D and 19E from *Serratia* strain AS13 showed statistical significance, killing more than 55% of *B. xylophilus* after 24 h (Figure 5, Table S6). Amino lipids 8C and 8D from *Serratia* strain Arv-22-2.5c, 9C from *Serratia* strain Arv-22-2.6, 17A, 17B and 17C from *Serratia* strain A88copa13, 18E, 18F and 18G from *Serratia* strain NBRC 102599^T^, 19D and 19E from *Serratia* strain AS13 were able to kill *B. xylophilus* after 48 h, with statistical significance. Negative controls 0.1 M NaCl solution and H_2_O were unable to kill more than 15% of *B. xylophilus*. The supernatant of *Serratia* strain A88copa13, a strain that produces a serine protease and that was used as a nematicidal positive control, was unable to kill more than 60% of nematodes.

### 2.5. Attraction Tests in C. elegans

Attraction tests are designed to demonstrate if young, starved *C. elegans* are more attracted to bacteria or supernatant of selected strains or to control *E. coli* OP50.

**Figure 6:**
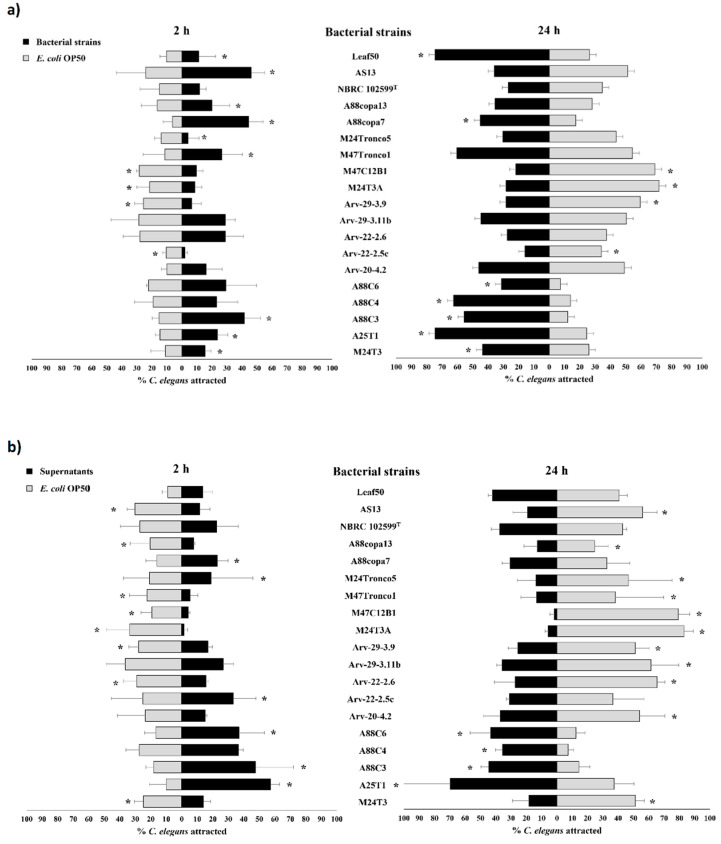

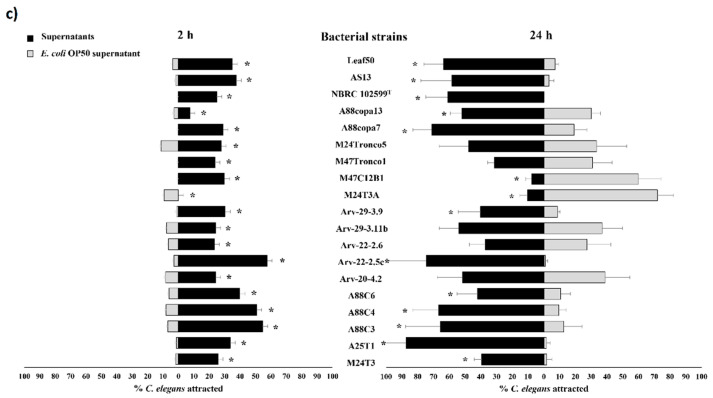
Attraction tests of *C. elegans* by bacterial suspensions and by their supernatants. The nematodes attraction was performed (**a**) by bacterial suspensions versus *E. coli* OP50 suspension as control; (**b**) by bacterial supernatants versus *E. coli* OP50 suspension as control; (**c**) by bacterial supernatants versus *E. coli* OP50 supernatant as control. All bacterial suspensions were used on attraction tests with an OD_600_ of 1.0. Bacterial supernatants were obtained after a 24 h growth. The number of attracted nematodes by bacteria and control was counted at 2 h and 24 h after *C. elegans* were added for all experiments. Each test was performed in triplicate. Results that showed statistical differences on chi-squared test, *p* < 0.05, are marked with (*). Bacterial strains: *Serratia* strains M24T3, A25T1, A88C3, A88C4, A88C6, Arv-20-4.2, Arv-22-2.5c, Arv-22-2.6, Arv-29-3.11b, Arv-29-3.9, M24T3A, M47C12B1, M24Tronco5, A88copa7, A88copa13, NBRC 102599^T^, AS13 and Leaf50, *Pseudomonas* strain M47Tronco1 and *E. coli* OP50. The lines above the bars represent standard deviation.

*Serratia* strains M24T3, A25T1, A88C3, A88copa7 and Leaf50 were able to attract more *C. elegans* than control after 2 h and maintained their attraction effect after 24 h, with statistical significance (Figure 6a, Table S7). *Pseudomonas* strain M47Tronco1 and *Serratia* strains A88copa13 and AS13 were able to attract more *C. elegans* than control after 2 h but lost attraction effect after 24 h, with statistical significance. *Serratia* strains A88C4 and A88C6 were not able to attract *C. elegans* more than control after 2 h, but were able to do it after 24 h, with statistical significance. *Serratia* strains Arv-22-2.5c, Arv-29-3.9, M24T3A and M47C12B1 were not able to attract *C. elegans*; in these cases, nematodes were more attracted to *E. coli* OP50 both after 2 h and after 24 h (Figure 6a).

Supernatants of Serratia strains A25T1, A88C3 and A88C6 were able to attract more *C. elegans* than the *E. coli* OP50 control after 2 h and after 24 h, with statistical significance. *Serratia* strains Arv-22-2.5c and A88copa7 attracted nematodes after 2 h with statistical significance but lost their ability to attract after 24 h. Nematodes were more attracted to *E. coli* OP50 control than to *Serratia* strains M24T3, Arv-20-4.2, Arv-22-2.6, Arv-29-3.9, M24T3A, M47C12B1, M24Tronco5, A88copa13, AS13 and *Pseudomonas* strain M47Tronco1 after 2 h and after 24 h (Figure 6b, Table S8).

All bacteria supernatants were able to attract more *C. elegans* than *E. coli* OP50 supernatant control at 2 h with two exceptions—strain M24T3A, for which nematodes were more attracted to *E. coli* OP50 supernatant, and strain M47C12B1, which was able to attract nematodes after 2 h but lost this ability after 24 h (Figure 6c, Table S9).

**Figure 7:**
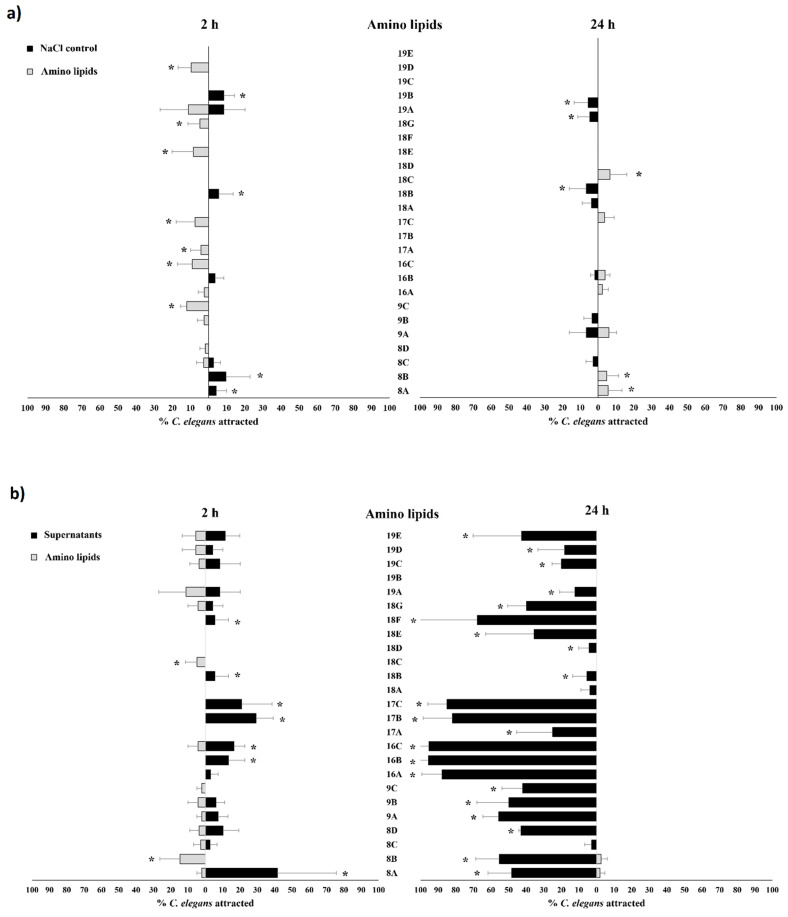

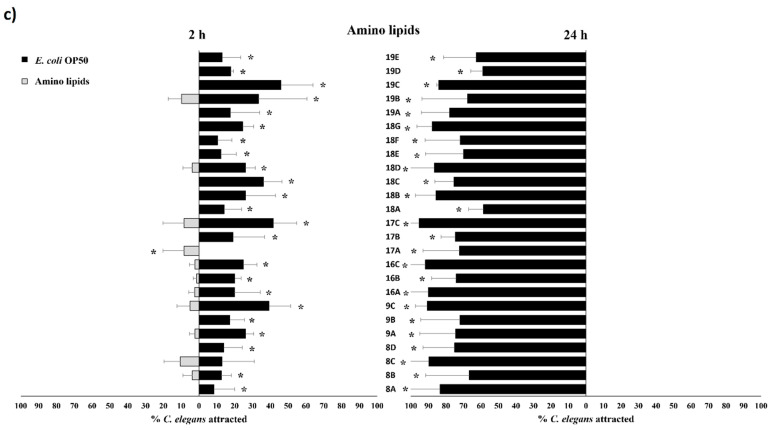
Attraction tests of *C. elegans* by amino lipids, by bacterial supernatants and *E. coli* OP50. The nematodes attraction was performed by (**a**) amino lipids eluted in 0.1 M NaCl versus 0.1 M NaCl solution as control; (**b**) by amino lipids eluted in 0.1 M NaCl versus bacterial supernatants; (**c**) by amino lipids eluted in 0.1 M NaCl versus *E. coli* OP50 suspension with an OD_600_ of 1.0. In all assays, amino lipids were extracted from supernatants of a 24 h growth of *Serratia* strains. The number of attracted nematodes by amino lipids, by bacterial supernatants and by *E. coli* OP50 was counted at 2 h and 24 h after *C. elegans* were added. Each test was performed in triplicate. Results that showed statistical differences on chi-squared test, *p* < 0.05, are marked with (*). *Serratia* strains and amino lipids—Arv-22-2.5c (amino lipids 8A, 8B, 8C and 8D), Arv-22-2.6 (amino lipids 9A, 9B and 9C), A88copa7 (amino lipids 16A, 16B and 16C), A88copa13 (amino lipids 17A, 17B and 17C), NBRC 102599^T^ (amino lipids 18A, 18B, 18C, 18D, 18E, 18F and 18G) and AS13 (amino lipids 19A, 19B, 19C, 19D and 19E). The lines above the bars represent standard deviation.

Attraction tests with extracted amino lipids against 0.1 M NaCl solution revealed a very low percentage of attraction (Figure 7a, Table S10). After 2 h, only amino lipids 9C from *Serratia* strain Arv-22-2.6, 16C from *Serratia* strain A88copa7, 17A and 17C from *Serratia* strain A88copa13, 18E and 18G from *Serratia* strain NBRC 102599^T^ and 19D from *Serratia* strain AS13 were able to attract more nematodes than control, with statistical significance. In attraction tests with amino lipids 8A and 8B from *Serratia* strain Arv-22-2.5c, 18B from *Serratia* strain NBRC 102599^T^ and 19B from *Serratia* strain AS13, nematodes preferred the NaCl solution. After 24 h, amino lipids 8A and 8B from *Serratia* strain Arv-22-2.5c and 18C from *Serratia* strain NBRC 102599^T^ were able to attract more nematodes than 0.1 M NaCl solution, with statistical significance. In attraction tests performed with amino lipids 18B and 18G from *Serratia* strain NBRC 102599^T^ and 19A from *Serratia* strain AS13, nematodes preferred the control solution.

Attraction tests performed with extracted amino lipids against supernatants revealed a poor attraction by amino lipids (Figure 7b, Table S11). After 2 h, only amino lipids 8B from *Serratia* strain Arv-22-2.5c and 18C from *Serratia* strain NBRC 102599^T^ were able to attract more nematodes than respective supernatants, with statistical significance. In attraction tests with amino lipids 8A from *Serratia* strain Arv-22-2.5c, 16B from *Serratia* strain A88copa7,17B and 17C from *Serratia* strain A88copa13, 18B and 18F from *Serratia* strain NBRC 102599^T^, nematodes preferred supernatants. After 48 h, almost all nematodes were attracted to supernatants, except for attraction tests with amino lipids 8C from *Serratia* strain Arv-22-2.5c, 18A and 18C from *Serratia* strain NBRC 102599^T^, and 19B from *Serratia* strain AS13, where most nematodes were dispersed over the NGM plate.

Attraction tests performed with extracted amino lipids against *E. coli* OP50 revealed that starved *C. elegans* preferred the food source over the amino lipids (Figure 7c, Table S12). After 2 h, only amino lipid 17A from *Serratia* strain A88copa13 was able to attract more nematodes than *E. coli* OP50, with statistical significance. After 24 h, nematodes were attracted by the food source in all tests.

### 2.6. HPLC Analysis of Selected TLC Bands

Eight extracted amino lipids were chosen for HPLC analysis since they showed better nematicidal activity against *B. xylophilus* when compared to other amino lipids. Amino lipids 8D from *Serratia* strain Arv-22-2.5c, 9C from *Serratia* strain Arv-22-2.6, 16C from *Serratia* strain A88copa7, 17C from *Serratia* strain A88copa13, 18F and 18G from *Serratia* strain NBRC 102599^T^ and 19D and 19E from *Serratia* strain AS13.

**Figure 8:**
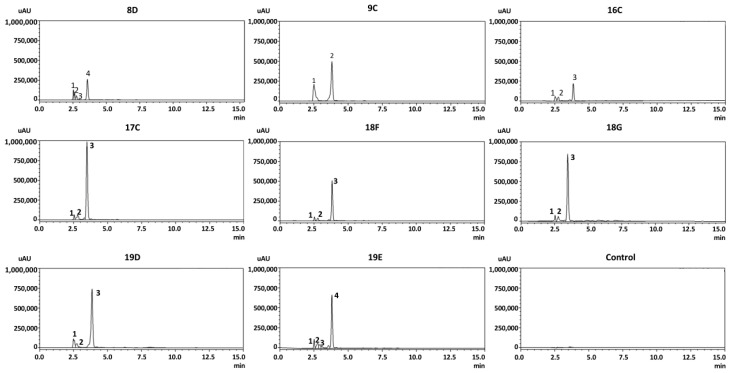
HPLC analysis of amino lipids produced by *Serratia* strains. Samples 8D from *Serratia* strain Arv-22-2.5c, 9C from *Serratia* strain Arv-22-2.6, 16C from *Serratia* strain A88copa7, 17C from *Serratia* strain A88copa13, 18F and 18G from *Serratia* strain NBRC 102599^T^ and 19D and 19E from *Serratia* strain AS13 and methanol control. HPLC was performed for 15 min with an eluent gradient of 87–92% of methanol. UV was detected at 215 nm.

HPLC analysis showed similar peaks in all amino lipids (Figure 8). All samples showed a peak between 3.48 and 3.86 min of retention time. Control with methanol reagent had no relevant peaks (Figure 8). High nematicidal activity of these extracted amino lipids may be associated with higher peaks.

## 3. Discussion

PWN, the known pathogenic agent of PWD, threatens pine and conifer forests all over the world. As global climate changes, a new scenario of PWN distribution evolves [36]. In order to prevent disease, spread, interactions between PWN and host trees, insect vectors and bacterial community must be understood. *Serratia* strains, previously described as part of the pine tree endophytic community [16] and associated with PWN [21,37], were chosen to study their nematicidal potential. This work intends to better understand the role of serratomolides, produced by *Serratia* strains, in PWD and, especially, to assess if they are effective against PWN. Serrawettin genes have already been found in several *Serratia* strains [30,32,33,34,35,38,39,40,41] and were present in seven of nineteen selected strains. The *swrW* gene was present in five *Serratia* strains and *swrA* gene was present in two *Serratia* strains. Serrawettin genes were also found in several *Serratia* strains resorting to computational approaches through AntiSMASH software [42]. Previously, several *Serratia* strains have been shown to produce high quantities of serrawettins, up to 17% of their mass dried weight [30]; some strains were already described as nematicidal [26,43]. The presence of other secondary metabolites described with nematicidal activity cannot be overruled [44].

Overall, the nematicidal effect of supernatants from selected strains was higher against *B. xylophilus* than against *C. elegans*. All supernatants were able to kill more than 30% of *B. xylophilus* after 48 h and more than 50% after 72 h, suggesting that this nematode is more susceptible to *Serratia* strains when compared to *C. elegans*. Our results confirm previous studies that identified some *Serratia* strains with nematicidal activity against *C. elegans* [33,45,46] and confirmed the nematicidal activity of *Serratia* strains against *B. xylophilus*, previously described [26]. It also expanded our knowledge about nematicidal activity of *Serratia* strains collected in different sampling sites, carried by PWN from USA [37] and from Portugal [21], and endophytic isolates from Portugal [16].

Concentrated supernatants presented better nematicidal results than non-concentrated supernatants and were able to kill more than 57% of *B. xylophilus* after 72 h. These results show that nematicidal activity increased as expected when supernatant components were more concentrated, which suggests that some extracellular components in *Serratia* supernatants possess strong nematicidal activity against nematodes. Concentrated supernatants from endophytic bacterial strains (*Serratia* strains A25T1, A88C4, A88C6), bacteria carried by PWN from Portugal (*Serratia* strain M24T3) and bacteria carried by PWN from USA (*Serratia* strains Arv-20-4.2, Arv-22-2.5c, Arv-22-2.6) were able to kill all *B. xylophilus* after 72 h. These results suggest that nematicidal activity is a characteristic that can be found in *Serratia* strains independently of the sample site. Mortality tests with concentrated supernatants and *C. elegans* revealed that *Serratia* strains A88C3, A88C4, A88C6, Arv-29-3.11b, Arv-29-3.9, A88copa7 and Leaf50, *Pseudomonas* strain M47Tronco1 and *E. coli* OP50 were able to kill all nematodes in 24 h. Even strains without *swrW* and *swrA* genes were able to produce high mortality rates, suggesting that nematicidal potential is not exclusively dependent on serratomolide presence, as observed for strains M24T3, A88C6 and A25T1. A previous study [26] had already concluded that some extracellular proteases, such as serine protease and serralysin, produced by *Serratia* strains, possess nematicidal activity against *B. xylophilus*.

Regarding attraction tests, 2 h results were considered more informative, as they had likely better reflected immediate attraction, whereas results after 24 h were probably more influenced by random nematode movements. *Serratia* strains M24T3, A25T1, A88C3, A88copa7 and Leaf50 were able to attract more nematodes than *E. coli* OP50 after 2 h and 24 h. Since *C. elegans* were starved, the preference for other bacteria than their usual food source reveals a strong attraction ability. Our findings confirm that *Serratia* strains have the ability to attract nematodes, as previously described [47] and that *C. elegans* is more attracted to some *Serratia* strains than to *E. coli* OP50, as previously described [46]. Supernatants of *Serratia* strains A25T1, A88C3, A88C4 and A88C6 were able to attract more nematodes than *E. coli* OP50 after 2 h and 24 h. None of these strains presented serratomolides genes, suggesting that another component in their supernatants is responsible for attraction. These results are in line with previous studies that showed that serrawettins produced by *Serratia* were able to act as repellent towards *C. elegans* [33] and showed nematostatic activity [48]. Some metabolites present in *Serratia* supernatants, such as amino acids, odors or autoinducers, may induce a food seeking behavior [33,49]. Almost all *Serratia* supernatants were able to attract more *C. elegans* than *E. coli* OP50 supernatant. This suggested that *C. elegans* may be attracted by some other *E. coli* OP50 cellular structure or component, that is absent or in low concentration in its respective supernatant, and/or that *C. elegans* preferred *Serratia* supernatant odors over *E. coli* OP50 odors, as previously described [50].

A total of 25 amino lipids from *Serratia* strains with *swrW* and *swrA* genes were extracted and purified from supernatants. Extracted amino lipids’ overall mortality rates against *C. elegans* were very low and identical to control. Mortality rates showed that *B. xylophilus* was more susceptible to these amino lipids than *C. elegans*. These results suggest that *B. xylophilus* may have more target proteins [51] susceptible to lipopeptides than *C. elegans*. Attraction tests with *C. elegans* revealed a poor attraction response to amino lipids and, overall, nematodes were more attracted to *Serratia* supernatants and food source *E. coli* OP50. As *C. elegans* were starved, these results suggest that amino lipids are not recognized as a potential food source. These results are consistent with the amino lipids’ absence in the nutritional requirements of *C. elegans* [52]. Amino lipids 8D, 9C, 16C, 17C, 18F, 18G, 19D and 19E had higher nematicidal activity against *B. xylophilus* than other extracted amino lipids from respective supernatants. These results seemed to indicate that, considering the mixture of amino lipids produced to the supernatant by the *Serratia* strains, the more polar amino lipids had more nematicidal activity against *B. xylophylus* and the less polar to *C. elegans*.

Subsequent HPLC analysis of these amino lipids extracted from *Serratia* strains with *swrW* and with *swrA* genes showed similar retention times on higher peaks, from 3.48–3.86 min. The results from this work indicated that amino lipids, serratomolides or other biosurfactants may be involved in *Serratia* nematicidal activity.

## 4. Materials and Methods

### 4.1. Bacterial Strains, Media and Growth Conditions

Twenty bacterial strains, 19 *Serratia* strains (seven strains isolated from Portuguese *Pinus pinaster* trees endophytic community [53], four PWN-carried strains from Portugal [21], five PWN-carried strains from USA [37] and three belonging to *S. plymuthica*) and one *Pseudomonas* strain carried by PWN from Portugal [21], used as control, were studied for lipopeptides serrawettin W1 and serrawettin W2 presence. Strains were grown on R2A agar medium for 24 h at 30 °C for DNA extraction. For mortality and attraction tests, bacterial strains were grown in Erlenmeyer’s flasks containing 50 mL of casamino acids (CAA) broth medium at 30 °C for 24 h, at 180 rpm [26]. Four mL of each bacterial growth were stored to be used on attraction tests. Bacterial growths were centrifuged at 12,100 rpm, at 4 °C, for 10 min, and 4 mL of each supernatant was used in mortality and attraction tests. Remainder supernatant was collected and concentrated with vacuum at −40 °C to be used on mortality tests.

### 4.2. DNA Extraction and Quantification

Genomic DNA extraction from all strains was performed using an NZY Microbial gDNA Isolation kit, according to manufacturer’s instructions. DNA samples were quantified using NanoDrop technology (Thermo Scientific^TM^, Waltham, MA, USA).

### 4.3. Primers Design, Polymerase Chain Reaction and DNA Sequence Analysis

To partially amplify *swrW* and *swrA* genes using PCR, new specific primers were designed (summarized in Tables S1 and S2). Eighty-five *Serratia* genomes available on NCBI were individually queried in the AntiSMASH database [42] to identify all secondary metabolite gene clusters (results published by authors [35]). Some of those secondary metabolite gene clusters presented DNA sequences similar to serrawettins’ genes: seventeen genomes with the *swrW* gene cluster and eleven with the *swrA* gene cluster [35]. Serratomolides genes were aligned and specific primers for *swrW* and *swrA* genes were designed, complementary to genetically conserved regions. Two primers for *swrW* gene were designed: swrW_1F, and swrW_7R. For the *swrA* gene, primers swrAA_1F and swrAA_10R were designed (Table S1).

In order to screen the presence of *swrW* and *swrA* genes in *Serratia* strains, PCRs were performed with selected primers. For each PCR, a mix was prepared containing, for each sample, 6.25 µL of MasterMix (NZYTaq II 2× Green Master Mix 0.2 U/μL), 0.2 µM of forward and reverse primers and 16.25 µL of ddH_2_O. One µL of DNA samples (Table S2) were added to a PCR tube containing the correspondent mix. For every mix, a negative control was made without DNA. PCR primers and gene length are summarized in Table S1. According to gene size and G+C mol% content of primers, temperature of annealing was adjusted. Electrophoreses were performed on 1% agarose gels at 90 V to separate and extract *swrW* and *swrA* genes. For PCR products that showed a band in the correspondent molecular size, DNA bands were removed and purified, using Omega’s Gel DNA Extraction Kit, according to the manufacturer’s instructions. DNA samples after gel extraction were sequenced by Stab Vida and the resulting sequences were compared with sequences available in EMBL/GenBank database using BLASTn network services [54].

### 4.4. Lipopeptides Extraction and Purification

*Serratia* strains Arv-22-2.5c, Arv-22-2.6, A88copa7, A88copa13, NBRC 102599^T^ and AS13, containing the *swrW* gene, were grown in 100 mL of R2A broth medium at 120 rpm and 30 °C with an initial OD_600_ of 0.07. After 24 h, OD_600_ was measured; cultures were centrifuged at 12,100 rpm for 30 min at 4 °C. Supernatants were transferred to clean frosted Erlenmeyers and were adjusted to pH 2 with a 12 M HCl solution. One hundred mL of a chloroform–methanol (2:1, *v/v*) solution was added to each supernatant and these were shook for 2 h. Organic phases were transferred, after phase separation, to clean frosted flasks [55]. Organic phases were concentrated using a rotary evaporator at 60 °C and residual liquid was concentrated with nitrogen. Crude extracts with lipids were resuspended with 2 mL of chloroform–methanol (2:1, *v/v*) solution. Polar lipids were separated from other lipids trough a column with 1 g of activated silica-gel 60. Columns were previously washed with 4 mL of methanol and 4 mL of a chloroform–methanol (9:1, *v/v*) solution before 2 mL of eluted crudes were passed through the silica column. Particles unable to bind to silica-gel were rescued on a flask. Apolar lipids were eluted with 7 mL of a chloroform–methanol (9:1, *v/v*) solution and polar lipids were eluted with 7 mL of methanol [55]. Flasks with polar lipid fractions were concentrated with nitrogen and resuspended in 250 µL of methanol. Purified polar lipids were examined with 1D TLC. TLCs were prepared in triplicate for each sample and were performed on silica 10 × 10 cm plates with 15 µL of each sample along 3 cm. Chloroform–acetic acid–methanol–water (80:15:12:4, *v/v*) solvent was used to run TLCs. When the run was concluded, TLC plates were dried, and ninhydrin reagent was sprayed on one of the three plates and stayed at 120 °C for 5 min to reveal amino lipids [56]. Positive spots with amino lipids were scraped from the other two plates without dye, and were extracted with 3 mL of chloroform–methanol (2:1, *v/v*) solution. After vortex and centrifugation at 13,300 rpm for 1 min in order to remove silica gel, chloroform–methanol (2:1, *v/v*) solutions with individualized polar lipids were transferred to clean flasks and evaporated with nitrogen. Extractions from one TLC plate were used for attraction and mortality tests on nematodes and were resuspended with 750 µL of 0.1 M NaCl solution. Assays regarding mortality tests in *C. elegans* and *B. xylophilus* and attraction tests in *C. elegans* were performed. The extracts obtained from a second TLC plate were used for HPLC analysis and were resuspended with 100 µL of methanol. HPLC was performed using an eluent gradient as previously described in [34], with some modifications. Eluent A was composed by a 0.05% trifluoracetic acid solution in purified water and eluent B was methanol. The eluent B gradient was 87–92% for 15 min. Flow rate was 1 mL/min and UV detection was measured at 215 nm. Methanol was used as control.

### 4.5. Maintenance of C. elegans

Nematodes were synchronized on nematode growth medium (NGM) agar plates with *E. coli* OP50 (source of food) twice a week in order to renew the medium and maintain young and adult nematodes. To synchronize *C. elegans*, NGM agar plates with nematodes in different stages were washed up and down with 2 mL of ddH_2_O. A fresh NaOH–bleach solution with 0.5 mL of NaOH (2 M) and 1 mL of 3% bleach was added to *C. elegans* suspension. After 10 min with vigorous vortex every 2 min, nematode suspension with dead nematodes and viable eggs was centrifuged at 13,000 rpm for 1 min, supernatant was discarded and pellet was washed with 1 mL of pure water, twice. Then, 150 µL of the final resuspended solution was distributed into new NGM plates with *E. coli* OP50. For attraction tests, *C. elegans* synchronization took place 24 h before the experiment in order to use stage L3 larvae in all tests. Attraction tests were performed with starved synchronized *C. elegans*: eggs hatched on NGM plates without *E. coli* OP50. For mortality tests, fed *C. elegans* were synchronized 48 h before tests [57]. For all experiments, Petri dishes with synchronized *C. elegans* were washed with 2 mL of ddH_2_O and the volume was adjusted in order to have a nematode suspension with 20–30 *C. elegans* on 10 µL for attraction tests and with 10 *C. elegans* on 10 µL for mortality tests.

### 4.6. Attraction Tests in C. elegans

Attraction tests were performed on NGM plates divided in three parts and equidistant spots. For each bacteria strain, three different attraction tests were made: (i) bacterial suspension on one side and *E. coli* OP50 on the other side as a control; (ii) supernatant of bacteria on one side and *E. coli* OP50 on the other side; (iii) and supernatant of bacteria on one side and supernatant of *E. coli* OP50 on the other side. Three attraction tests were performed with *C. elegans* and amino lipids: (i) with amino lipids versus 0.1 M NaCl solution; (ii) with amino lipids versus respective supernatant; (iii) and with amino lipids versus *E. coli* OP50. With attraction tests, we assessed the percentage of nematodes drawn to bacteria, supernatants and extracted amino lipids. For bacterial suspension, 10 µL with OD_600_ = 1.0 were placed on the correspondent side of NGM agar plates. 10 µL of supernatants obtained by bacterial growth centrifugation and 10 µL of eluted amino lipids were used. After 30 min, for drops to dry, 10 µL of a nematode suspension, with approximately 20–30 *C. elegans*, was added to the third side of the NGM plate. The initial number of *C. elegans* in every plate was counted. Each attraction test was performed in triplicate. All NGM plates were sealed with Parafilm and were put at 19 °C. After 2 h and 24 h, nematodes on each drop were assessed to see if bacteria or supernatants were able to attract more nematodes than control [47,58].

### 4.7. Mortality Tests in C. elegans and B. xylophilus

Mortality tests were performed in 24 multi-well plates for both *C. elegans* and *B. xylophilus*. For *C. elegans*’ mortality tests, 10 µL of a nematode suspension with approximately 10 *C. elegans* was inserted in the middle of the well. The initial number of *C. elegans* in each well was assessed. For *B. xylophilus*’ mortality tests, 50 µL of ddH_2_O was put on the middle of the well and, for each well, exactly 10 young *B. xylophilus* were added using a disinfected eyelash. Every test was performed in triplicate with 500 µL of concentrated bacterial supernatants and once with supernatants. After 24 h, 48 h and 72 h, dead nematodes were assessed [26]. CAA medium (bacterial growth medium) and M9 medium (liquid medium used on *C. elegans* maintenance) were used as negative controls. To assess which amino lipid had a greater nematicidal effect, dead nematodes percentage was determined after a period of 24 h and 48 h with 500 µL of amino lipid suspension in NaCl solution [26].

### 4.8. Statistical Analysis

In order to find statistical differences among mortality test results, a one-way ANOVA was performed, separately, with data of mortality tests obtained from *B. xylophilus* and *C. elegans*, for each time point using the exposure treatment (strain or amino lipid) as a factor for comparison. Subsequently, when significant differences were detected by one-way ANOVA, a pairwise comparison was performed using the Tukey’s test. Attraction test results were analyzed using a Chi-Square statistical test to detect significant differences in the nematodes’ proportions between (i) bacterial suspensions and *E. coli* OP50 suspension as control; (ii) bacterial supernatants and *E. coli* OP50 suspension as control; (iii) bacterial supernatants and *E. coli* OP50 supernatant as control; (iv) amino lipids eluted in 0.1 M NaCl and 0.1 M NaCl solution as control; (v) amino lipids eluted in 0.1 M NaCl and corresponding bacterial supernatants; and (vi) amino lipids eluted in 0.1 M NaCl and *E. coli* OP50 suspension. All statistical analyses were performed using R packages stats (v4.1.2) and dplyr (v1.0.7) in R software (v4.1.2, https://www.r-project.org/, accessed on 15 December 2021) [59] and results were considered statistically different when *p <* 0.05.

### 4.9. Nucleotide Sequence Accession Numbers

The *swrW* and *swrA* gene sequences of the bacterial isolates reported in this study were deposited at the Genbank database, under the accession numbers OK500210-OK500216.

## Figures and Tables

**Table 1:** Sequencing and BLASTn results of *swrW* and *swrA* partial genes sequences of Serratia isolates.

Bacterial Strain	PCR Code	PCR Product Length	Accession Number	*Serratia* Species	Gene	Identity %	Cover %	Accession Number
*Serratia* sp. Arv-20-4.2	8	555	OK500210	*Serratia marcescens*	*swrW*	98.38%	100%	AB193098
*Serratia* sp. Arv-22-2.5c	9	438	OK500211	*Serratia marcescens*	*swrW*	98.87%	100%	AB193098
*Serratia* sp. Arv-29-3.11b	11	447	OK500212	*Serratia marcescens*	*swrW*	98.88%	100%	AB193098
*Serratia* sp. NBRC 102599^T^	18	430	OK500213	*Serratia marcescens*	*swrW*	72.52%	100%	AB193098
*Serratia* sp. AS13	19	452	OK500214	*Serratia marcescens*	*swrW*	73.46%	96%	AB193098
*Serratia* sp. A88copa7	16	357	OK500215	*Serratia liquefaciens*	*swrA*	71.27%	94%	AF039572
*Serratia* sp. A88copa13	17	455	OK500216	*Serratia liquefaciens*	*swrA*	69.36%	99%	AF039572

## Data Availability

The data presented in this study are available within this article and in supplementary material.

## Supplementary Materials

The following supporting information can be downloaded at: https://www.mdpi.com/article/10.3390/pathogens11020198/s1, Table S1: Primers information to test the presence of *swrW* gene (PCR A) and *swrA* gene (PCR C); Table S2: Bacterial DNA concentrations of 19 *Serratia* strains M24T3, A25T1, A52T1, A88C3, A88C4, A88C6, Arv-20-4.2, Arv-22-2.5c, Arv-22-2.6, Arv-29-3.11b, Arv-29-3.9, M24T3A, M47C12B1, M24Tronco5, A88copa7, A88copa13, NBRC 102599^T^, AS13 and Leaf50 and *Pseudomonas* strain M47Tronco1; Table S3: Optical density of 24 h growth of *Serratia* strains Arv-22-2.5c, Arv-22-2.6, A88copa7, A88copa13, NBRC 102599^T^ and AS13; Table S4: Mean and standard deviation of *C. elegans*’ mortality tests (%) with bacterial strains and controls M9 and CAA media. Tukey’s test *p* values for each strain versus control CAA medium are given for each time point 24, 48 and 72 h; Table S5: Mean and standard deviation of *Bursaphenchus xylophilus*’ mortality tests (%) with concentrated supernatants of bacterial strains and controls M9 and CAA media. Tukey’s test *p* values for each strain versus control CAA medium are given for each time point 24, 48 and 72 h; Table S6: Mean and standard deviation of *B. xylophilus*’ mortality tests (%) with amino lipids eluted in 0.1 M NaCl solution and controls H_2_O and 0.1 M NaCl solution. Tukey’s test *p* values for each amino lipid versus control 0.1 M NaCl solution are given for each time point 24 and 48 h; Table S7: Statistical analysis of attraction tests of *C. elegans* by bacterial suspensions versus *E. coli* OP50 suspension as control, using the mean proportion of nematodes as the dependent variable. Chi-square test value and the associated *p* value are given for *C. elegans*’ attraction tests at 2 and 24 h; Table S8: Statistical analysis of attraction tests of *C. elegans* by bacterial supernatants versus *E. coli* OP50 suspension as control, using the mean proportion of nematodes as dependent variable. Chi-square test value and the associated *p* value are given for *C. elegans*’ attraction tests at 2 and 24 h; Table S9: Statistical analysis of attraction tests of *C. elegans* by bacterial supernatants versus *E. coli* OP50 supernatant as control, using the mean proportion of nematodes as dependent variable. Chi-square test value and the associated *p* value are given for *C. elegans*’ attraction tests at 2 and 24 h; Table S10: Statistical analysis of attraction tests of *C. elegans* by amino lipids eluted in 0.1 M NaCl versus 0.1 M NaCl solution as control, using the mean proportion of nematodes as dependent variable. Chi-square test value and the associated *p* value are given for *C. elegans*’ attraction tests at 2 and 24 h; Table S11: Statistical analysis of attraction tests of *C. elegans* by amino lipids eluted in 0.1 M NaCl versus corresponding bacterial supernatants, using the mean proportion of nematodes as dependent variable. Chi-square test value and the associated *p* value are given for *C. elegans*’ attraction tests at 2 and 24 h; Table S12: Statistical analysis of attraction tests of *C. elegans* by amino lipids eluted in 0.1 M NaCl versus *E. coli* OP50 suspension, using the mean proportion of nematodes as dependent variable. Chi-square test value and the associated *p* value are given for *C. elegans*’ attraction tests at 2 and 24 h.
