# Supplementary material for: The Role of Serratomolide-like Amino Lipids Produced by Bacteria of Genus Serratia in Nematicidal Activity
Source: Pathogens. 2022 Feb 1;11(2):198. doi: 10.3390/pathogens11020198 (PMC8880026; doi:10.3390/pathogens11020198)
Supplement: Supplementary file 1 [file pathogens-11-00198-s001.zip › pathogens-1422785-supplementary.pdf]

pathogens

MDPI

Article

# The Role of Serratomolide-Like Amino Lipids Produced by Bacteria of Genus *Serratia* in Nematicidal Activity

**Catarina Marques-Pereira[1†], Diogo Neves Proença[1*], Paula V. Morais[1]**

Department of Life Sciences, Centre for Mechanical Engineering, Materials and Processes, University of Coimbra, Calçada Martim de Freitas, 3000-456 Coimbra, Portugal; catarina.103@gmail.com (C.M.-P.); pvmorais@uc.pt (P.V.M.)

* Correspondence: diogo.proenca@uc.pt; Tel.: +351-239240700

† Current address: Center for Neuroscience and Cell Biology, Center for Innovative Biomedicine and Biotechnology, University of Coimbra, 3004-504 Coimbra, Portugal.

## Supplementary material

**Table S1.** Primers information to test the presence of *swrW* gene (PCR A) and *swrA* gene (PCR C).

| Primer | Temperature cycling (30x) | Primer sequence | Total Length (bp) |
|---|---|---|---|
| PCR A | 95 ºC for 60 s | *swrW*_1F: 5′-GTGTCCGCTTATTCYCTSAC-3′ | 3,037 |
| | 56 ºC for 60 s | *swrW*_7R: 5′-TGAATGGCRTGCAGCGAATG-3′ | |
| | 72 ºC for 210 s | | |
| PCR C | 95 ºC for 60 s | *swrA*A_1F: 5′-ATGAACAAACAMACTGATGTG-3′ | 2,652 |
| | 56 ºC for 60 s | *swrA*A_10R: 5′-CCGCGCCTGCGCTTCRAACAG-3′ | |
| | 72 ºC for 180 s | | |

**Table S2.** Bacterial DNA concentrations of 19 *Serratia* strains M24T3, A25T1, A52T1, A88C3, A88C4, A88C6, Arv-20-4.2, Arv-22-2.5c, Arv-22-2.6, Arv-29-3.11b, Arv-29-3.9, M24T3A, M47C12B1, M24Tronco5, A88copa7, A88copa13, NBRC 102599$^{T}$, AS13 and Leaf50 and *Pseudomonas* strain M47Tronco1.

| Bacterial strains | ng/µL |
|---|---|
| M24T3 | 117.7 |
| A25T1 | 65.9 |
| A52T1 | 1.5 |
| A88C3 | 190.6 |
| A88C4 | 104.3 |
| A88C6 | 179.3 |
| Arv-20-4.2 | 128.8 |
| Arv-22-2.5c | 60.6 |
| Arv-22-2.6 | 181.4 |
| Arv-29-3.11b | 106.1 |
| Arv-29-3.9 | 117.1 |
| M24T3A | 51.3 |
| M47C12B1 | 62.0 |
| M47Tronco1 | 40.6 |
| M24Tronco5 | 160.4 |
| A88copa7 | 172.9 |
| A88copa13 | 167.6 |
| NBRC 102599$^{T}$ | 39.3 |
| AS13 | 82.6 |
| Leaf50 | 53.4 |

**Table S3.** Optical density of a 24 h growth of *Serratia* strains Arv-22-2.5c, Arv-22-2.6, A88copa7, A88copa13, NBRC 102599$^{T}$ and AS13.

| *Serratia* strains | $OD_{600}$ |
|---|---|
| Arv-22-2.5c | 1.21 |
| Arv-22-2.6 | 1.12 |
| A88copa7 | 0.93 |
| A88copa13 | 1.73 |
| NBRC 102599$^{T}$ | 0.65 |
| AS13 | 0.73 |

**Table S4.** Mean and standard deviation of *C. elegans´* mortality tests (%) with bacterial strains and controls M9 and CAA media. Tukey´s test *p* values for each strain versus control CAA medium are given for each time point 24, 48 and 72 h.

(In excell file).

**Table S5.** Mean and standard deviation of *Bursaphenchus xylophilus´* mortality tests (%) with concentrated supernatants of bacterial strains and controls M9 and CAA media. Tukey´s test *p* values for each strain versus control CAA medium are given for each time point 24, 48 and 72 h.

(In excell file).

**Table S6.** Mean and standard deviation of *B. xylophilus´* mortality tests (%) with amino lipids eluted in 0.1 M NaCl solution and controls $H_2O$ and 0.1 M NaCl solution. Tukey´s test *p* values for each amino lipid versus control 0.1 M NaCl solution are given for each time point 24 and 48 h.

(In excell file).

**Table S7.** Statistical analysis of attraction tests of *C. elegans* by bacterial suspensions versus *E. coli* OP50 suspension as control, using the mean proportion of nematodes as dependent variable. Chi-Square test value and the associated *p* value are given for *C. elegans´* attraction tests at 2 and 24 h.

(In excell file).

**Table S8.** Statistical analysis of attraction tests of *C. elegans* by bacterial supernatants versus *E. coli* OP50 suspension as control, using the mean proportion of nematodes as dependent variable. Chi-Square test value and the associated *p* value are given for *C. elegans´* attraction tests at 2 and 24 h.

(In excell file).

**Table S9.** Statistical analysis of attraction tests of *C. elegans* by bacterial supernatants versus *E. coli* OP50 supernatant as control, using the mean proportion of nematodes as dependent variable. Chi-Square Test value and the associated *p* value are given for *C. elegans´* attraction tests at 2 and 24 h.

(In excell file).

**Table S10.** Statistical analysis of attraction tests of *C. elegans* by amino lipids eluted in 0.1 M NaCl versus 0.1 M NaCl solution as control, using the mean proportion of nematodes as dependent variable. Chi-Square test value and the associated *p* value are given for *C. elegans´* attraction tests at 2 and 24 h.

(In excell file).

**Table S11.** Statistical analysis of attraction tests of *C. elegans* by amino lipids eluted in 0.1 M NaCl versus corresponding bacterial supernatants, using the mean proportion of nematodes as dependent variable. Chi-Square test value and the associated *p* value are given for *C. elegans´* attraction tests at 2 and 24 h.

(In excell file).

**Table S12.** Statistical analysis of attraction tests of *C. elegans* by amino lipids eluted in 0.1 M NaCl versus *E. coli* OP50 suspension, using the mean proportion of nematodes as dependent variable. Chi-Square test value and the associated *p* value are given for *C. elegans´* attraction tests at 2 and 24 h.

(In excell file).
